# The Swedish model of health dialogues, a combined individual- and community-based primary preventive program for cardiovascular disease, is associated with reduced mortality: a systematic review

**DOI:** 10.1186/s12889-025-24353-0

**Published:** 2025-10-02

**Authors:** Mats Borjesson, Margareta Kristenson, Lars Jerdén, Yvonne Forsell

**Affiliations:** 1 Institution of Medicine, Department of Molecular and Clinical Medicine , Sahlgrenska Academy, University of Gothenburg, Gothenburg, Sweden; 2 Department of Medicine, Geriatrics and Emergency Care, Center for Lifestyle Intervention, Sahlgrenska University Hospital, Gothenburg, Sweden; 3 Department of Health, Medicine and Caring Sciences, Linköping University, Linköping, Sweden; 4 Center for Clinical Research Dalarna-Uppsala University, Uppsala, Sweden; 5 Department of Global Public Health, Karolinska Institute, Stockholm, Sweden

**Keywords:** Systematic Review, Life Style, Primary Prevention, Counseling, Cardiovascular Disease, Smoking

## Abstract

**Background:**

Behavioural risk factors are key determinants of premature death. The Swedish model of health dialogues, which has been developed since 1985, aims to promote healthy behaviours, for prevention of cardiovascular disease, by inviting everyone in specific age groups to primary care for health dialogues combined with community-oriented activities. The health dialogue was performed by trained nurses, based on individual results from questionnaires on health behaviors and results of physiological measures, using visual pedagogic tools and motivational interviewing techniques. The community intervention part of the program aimed to encourage and enhance healthy behaviors, e.g. by collaboration with civil society, e.g. local sports organizations and/or grocery stores.

**Methods:**

In this first systematic review of the model, seven studies were identified fulfilling the inclusion criteria, having control/reference groups, defined outcome health measures (mortality, risk factors, lifestyle behaviours) and follow-ups of at least one year. GRADE (Grading of Recommendations, Assessment, Development and Evaluation) was used to assess the quality of evidence. The effect on mortality, on risk factors and lifestyle behaviours was evaluated.

**Results:**

The model showed effects with reduced premature all-cause mortality and cardiovascular mortality with a moderate level of evidence. Specifically, intention to treat analyses showed a 9.4% and 29% reduction in all-cause mortality in two studies, and 5% reduction of cardiovascular mortality in one large study. Furthermore, levels of blood pressure, cholesterol, fasting blood glucose, waist and BMI were reduced (moderate/low level of evidence) and dietary habits were improved (moderate level of evidence). Health benefits of the model were greater when health dialogues were combined with community-oriented activities.

**Conclusions:**

The results of this first systematic review of the Swedish model of health dialogues showed significant effects of the Swedish model of health dialogue, on cardiovascular and all-cause mortality. The method is a multifactorial intervention that includes both individual and community/societal intervention, where the respective contributions of both parts of the intervention cannot be delineated.

## Introduction

According to the World Health Organization (WHO), up to 80% of cases of myocardial infarction and stroke can be prevented by addressing behavioral risk factors such as tobacco use, unhealthy diet, physical inactivity, and harmful use of alcohol [1]. In Sweden, as in many other Western European countries, the cardiovascular disease (CVD)-mortality has decreased in recent decades, which is mainly due to the reduction of some risk factors, including smoking [2]. However, CVD is still the leading cause of premature death [3], constituting a major public health problem.

National guidelines for prevention and treatment of behavioral risk factors, were published by the Swedish National Board of Health and Welfare in 2011, with the latest update in 2024 [4]. However, the focus of these guidelines is on patients, and most interventions therefore are secondary, i.e. applied to those with already established disorders related to health behaviors, e.g. CVD, diabetes. Given that 60–70% of deaths within 28 days after acute myocardial infarction occurred before hospitalization [5], primary prevention is also clearly needed.

In primary preventive work, a common aim is to identify people at highest risk, assuming that this would be the most effective strategy. However, as described by Rose [6], because the population with medium risk is much larger, the majority of new patients and burden of disease can be attributed to individuals in this lower risk group. To reduce the population disease burden, the “prevention paradox” states that we need to apply a population strategy, in addition to the high-risk strategy, aimed at moving the distribution of risk factors towards lower levels. To achieve this, there is a need to target different levels: individuals, populations and structural factors [5].

**Fig. 1:**
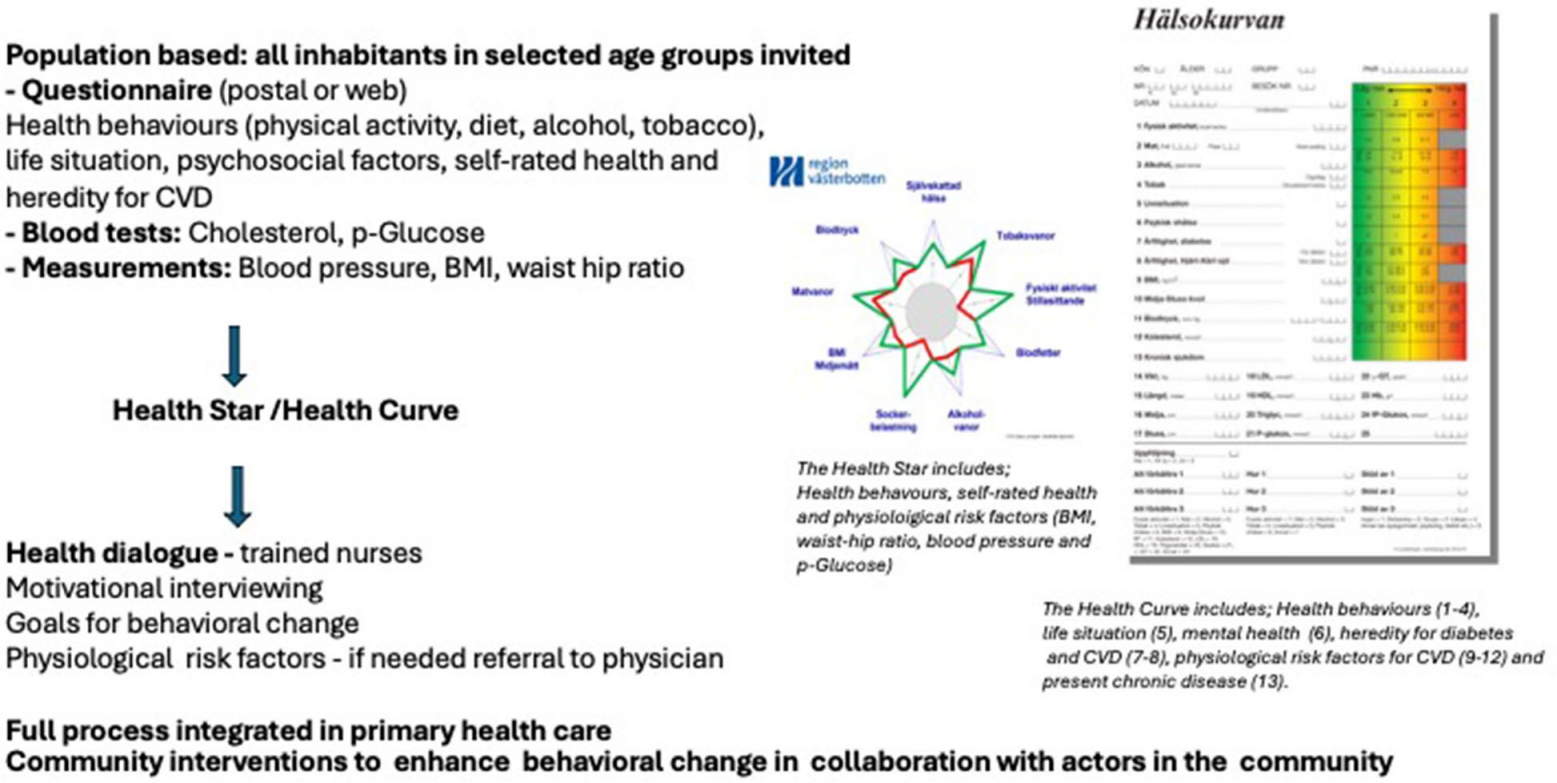
Swedish model of health dialogues. The full process, integrated in primary care, includes target population, tests/measurements, Health Star/Health Curve, Health dialogue and Community interventions

In 1985 a Swedish model of health dialogues combined with a community-based program, targeting CVD, was developed in two regions: Skaraborg/Jönköping in southern Sweden and Västerbotten in the northern part of Sweden. The aim was to develop a program for primary prevention of CVD by targeting behavioral and biological risk factors in the general population. In the individual part of the program, all persons in selected age-groups (e.g. turning 40, 50 and 60 years) were invited to the local primary care units for examinations and a health dialogue. The health dialogue was performed by trained nurses, based on individual results from questionnaires on health behaviors (physical activity, diet, alcohol, and tobacco use), psychosocial factors, socioeconomic status (SES), heredity for CVD, and results of physiological measures (blood pressure, blood lipids, blood sugar, weight, abdominal fat) (Fig. 1). These dialogues were performed using visual pedagogic tools (a Star or a Health Curve illustrating individuals risk profile, see Fig. 1) and motivational interviewing techniques. The community intervention part of the program aimed to encourage and enhance healthy behaviors, e.g. by collaboration with civil society, e.g. local sport organizations and/or grocery stores.

For comparison, General health checks have been used in various countries since the early 1960s. These examinations are often extensive screening for latent disease and risk factors, with the aim of early detection of disease or to give reassurance. A Cochrane report on effects of general health checks [7] concluded that they had no effect on all-cause mortality or on incidence of CVD or cancer. Another Cochrane report [8], which focused on effects of general health checks that target risk factors for CVD, identified effects on mortality and morbidity among individuals with hypertension and diabetes but not in the general population. Differing from health dialogues, only 5 out of 17 studies were performed in primary care, and only a few included advice for behavioral change, and none of the interventions in these reports included community-oriented activities.

To support the implementation of the national guidelines on promoting healthy behaviours, practical recommendations were developed in 2022 by the National system for knowledge-driven healthcare in Sweden. This work identified a need for evaluation of the existing evidence for the Swedish model for health dialogues. Therefore, an expert group, the authors of this paper, was appointed, with the aim to review the effects of the model.

This systematic review reports on the evidence of the effect of interventions according to the Swedish model of health dialogues on all-cause and CVD mortality, and on risk factors, including behavioral factors. A lengthy report from the review [9], and a shorter paper in the Läkartidningen [10] have been published in the Swedish language.

## Methods

The evaluation included a systematic literature search followed by a classification and evaluation of individual studies and of the evidence for the effect of the interventions on different outcomes (all-cause and CVD mortality, morbidity and risk factors) using the GRADE system (Grading of Recommendations Assessment Development and Evaluation) [11].

### Literature search

A literature search was performed using the databases PubMed, Cinahl, PsycInfo and SweMed with the following search criteria: ("health communication"OR"health dialogue"OR"health check") AND ("primary health care") AND (Sweden OR Swedish).

The following criteria were used for selection of studies:

- Outcomes: All-cause and CVD mortality; CVD morbidity (registered diagnoses); diagnosis of diabetes; biological measurements: systolic and diastolic blood pressure, total serum cholesterol, BMI, waist circumference and fasting blood glucose; and health behaviors: use of tobacco or alcohol, physical activity, and diet.
- Additional criteria: At least one-year follow-up. Some form of control/reference group should be included.
- Context: Integrated in a Swedish primary care setting and performed in a context of societal interventions aimed at facilitating improvements of health behaviors.
- Population: All inhabitants from selected age groups in the general population. The age groups differed between the studies, the youngest invited age group was 30 years (12-13, 16), the oldest 60 years (12-15).
- Intervention**:** Interventions should include invitations to individual health dialogues with primary focus on CVD or diabetes (including relevant risk factors), and additionally a community-based intervention to enhance healthy behaviours.

Studies were initially identified, by searches on PubMed (*n* = 21); Cinahl (*n* = 5) (all of which were also present in PubMed); PsycInfo (*n* = 12, of which 7 were not present in PubMed) and SweMed (*n* = 20, none of which were present in PubMed), totalling 48 unique studies.

**Fig. 2:**
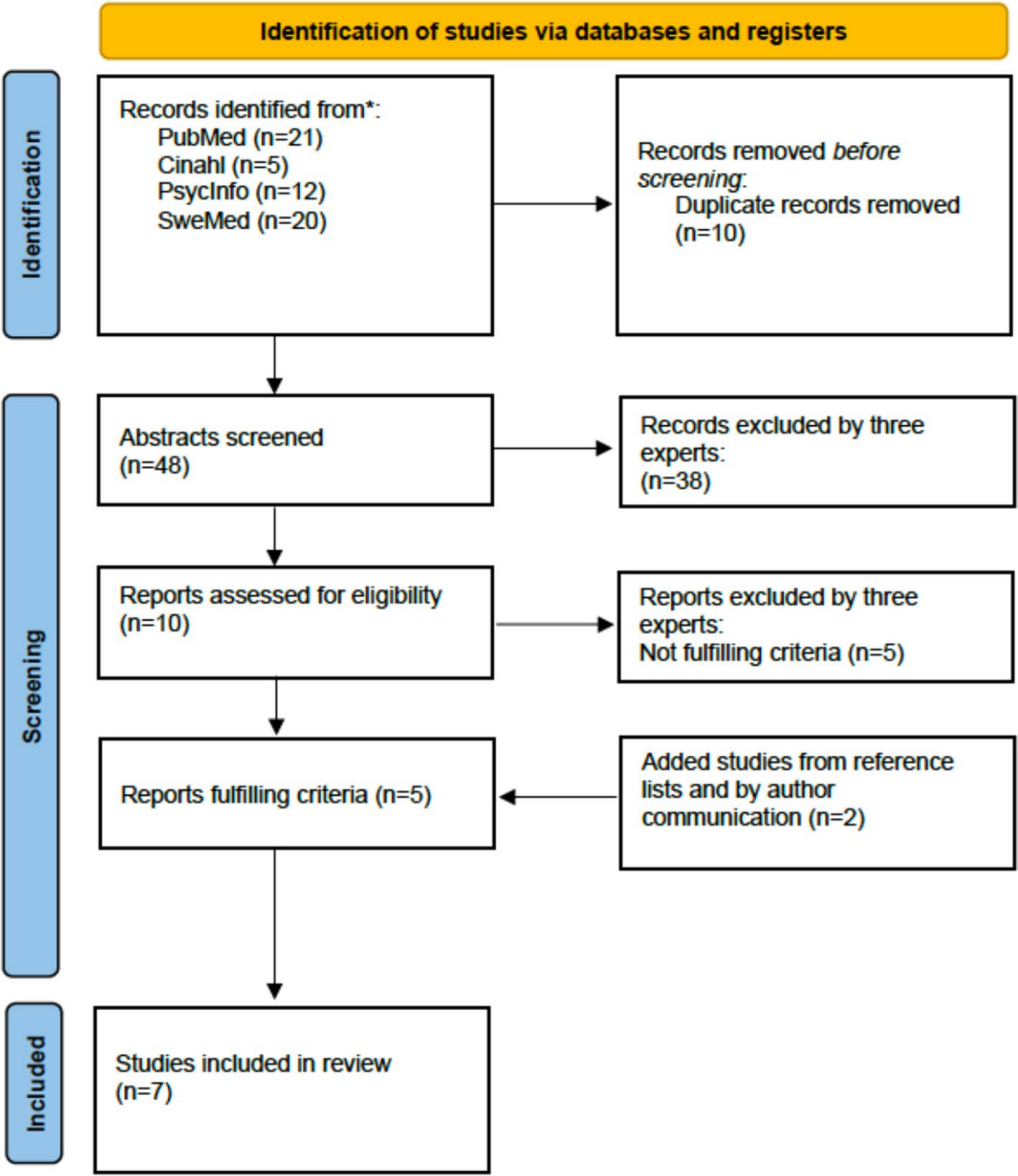
PRISMA flow diagram of search of databases and study inclusion

All abstracts were read carefully, by three independent experts in the field (two of the authors and an external) and ten papers were deemed as relevant and were scrutinized in detail. Five of these fulfilled the criteria to be included in the review. By scrutinizing the reference lists and by personal contact to the authors, two more studies fulfilling the critera, were identified. In total, seven papers were included in the final review [12–18] (Fig. 2).

None of the papers had studied any other tobacco-related outcome, apart from smoking (i.e. snuff) and no study had cardiovascular morbidity as an outcome. All included studies were performed in three Swedish regions. Six were observational, non-randomized controlled studies (NRCT) from Västerbotten, Skaraborg/Jönköping, and one was a randomized controlled trial (RCT) from Västmanland. The full study characteristics of the included studies are presented in Tables 1 and 2.

**Table 1:** Study characteristics: design, setting, participants and outcome measures

**Study**	**Design** I: intervention C: reference/control	**Setting, Participants** I: intervention C: reference/control	**Outcomes**
Weinehall et al. Shifting the distribution of risk J Epid Comm Health. 1999;53:243–50 [12]	Prospective study comparing cross sections during 8 years (I) and 4 years (C)	I: Norsjö municipality *n* = 1 893 Ages: 30, 40, 50, 60 years C: Regions of Norr- and Västerbotten (from the WHO MONICA study*) *n* = 3 208 Ages: 25–64 years	Cholesterol Systolic blood pressure Diastolic blood pressure Body Mass Index Smoking
Weinehall et al. Can a sustainable community intervention reduce the health gap? Scand J Public Health Suppl 2001;56:59–68 [13]	Prospective study of repeated independent cross-sectional surveys over 10 years	I: Norsjö municipality *n* = 2 289 Ages: 30, 40, 50, 60 years C: Norr- and Västerbotten regions (from the WHO MONICA study*) *n* = 4 749 Ages: 25–64 years	Cholesterol Systolic blood pressure Diastolic blood pressure Smoking
Lingfors et al. Effects of a global health and risk assessment tool for prevention of ischemic heart disease in an individual health dialogue compared with a community health strategy only Prev Med 2009;48:20–4 [16]	A cohort in the intervention area compared to two cross-sections in the control area Follow up during 5 years	I: 4 municipalities Skaraborg region (offered health dialogues at 30 and 35 years) *n* = 1 469 Age: 30 years C: 4 municipalities Skaraborg region (offered health dialogues at 35 years) *n* = 3 309 Age: 35 years	Cholesterol Systolic blood pressure Diastolic blood pressure Body Mass Index Waist measurement Physical activity Smoking Eating habits
Blomstedt et al. Impact of a combined community and primary care prevention strategy on all-cause and cardiovascular mortality: a cohort analysis. BMJ Open 2015;5:e009651 [14]	Prospective cohort study Follow-up in average 10 years	I: Västerbotten region *n* = 101 918 Ages: 40, 50, 60 years C: Sweden (registers from the National Board of Health and Welfare, and Statistics Sweden) *N* = 3 472 164 Ages: 40, 50, 60 years	All-cause mortality Cardiovascular mortality
Hellstrand et al. A health dialogue intervention reduces cardiovascular risk factor levels BMC Public Health 2017;17:669 [18]	Randomized controlled study Follow-up 1 year	I: Västmanland region *n* = 231 Age: 55 years C: Västmanland region *n* = 229 Age: 55 years	Cholesterol Fasting P-glucose Systolic blood pressure Diastolic blood pressure Body Mass Index Waist measurement Physical activity Smoking Eating habits Alcohol consumption
Eliasson et al. Comparison of trends in cardiovascular risk factors between two regions with and without a community and primary care prevention programme. Eur J Prev Cardiol 2018:25:1765–72 [15]	Prospective study of repeated cross-sections during 20 years	I: Västerbotten region *n* = 3 181 Ages: 40–75 years C: Norrbotten region (both I and C from the WHO MONICA study**) *n* = 3 419 Ages: 40–75 years	Cholesterol Fasting P-glucose Diabetes Systolic blood pressure Diastolic blood pressure Body Mass Index Waist measurement Physical activity Smoking
Lingfors et al. All-cause mortality among young men 24–26 years after a lifestyle health dialogue in a Swedish primary care setting. BMJ Open 2019;9:e022474 [17]	Prospective cohort study Follow-up 26 years	I: Habo municipality *n* = 757 Ages: 33–42 years C: Sweden (registers from the National Board of Health and Welfare) *N* = 656 686 Ages: 33–42 years	All-cause mortality

^*^Reference [11] in Weinehall et al. 1999

^**^References [5] and [8] in Eliasson et al. 2017

**Table 2:** Study characteristics: interventions and transferability

Study	Health dialogues		Community oriented measures		Transferability
	Number offered intervention group	Proportion of invited who attended dialogues (%)	Intervention	Reference/control	Downgrading of level of evidence because of transferability*
Weinehall 1999 [12]	1–2	92.5	Very extensive	Limited	−1
Weinehall 2001** [13]	1	> 90	Very extensive	Limited, except 1994 in Västerbotten (extensive)	−1
Lingfors 2009*** [16]	2	58–64	Extensive	Extensive	0
Blomstedt 2015 [14]	1–3	59	Extensive	Limited	0
Hellstrand 2017**** [18]	2	53	Limited	Limited	0
Eliasson 2018 [15]	1–3	Data not available	Extensive	Limited	0
Lingfors 2019 [17]	1–2	86	Very extensive	Limited	−1

^*^Downgrading one level (−1) because of risk of insufficient transferability/external validity when the community-oriented measures had been very extensive

^**^In the reference area, some of the participants in Västerbotten had been offered a health dialogue in 1994

***The intervention group were offered healthdialogues at the ages of 30 and 35 years, while the control group only wereoffered the health dialogue at the age of 35

****The second health dialogue was a follow-up after 1 year

### Evaluation of level of evidence

GRADE classifies studies into high + + + +, moderate + + +, low + +, or very low + evidence, based on evaluations of five domains (see below). The starting point for every study is high evidence, which can then be modified according to possible limitations in these domains [19].

The included studies were, thus, classified based on each of the following five domains, according to their quality of design.

1. Risk of bias (*confounding, selection, classification, interventions, missing data, measurement of outcomes, and reported results).*
2. Inconsistency (heterogenicity)
3. Transferability
4. Imprecision
5. Publication bias

If serious problems in one of these domains were identified, the level of evidence was downgraded one level. The level of grading could also be upgraded (one level) if a large effect size or large dose–response relationship was demonstrated, but only if no serious problems were observed.

### Extent of intervention and transferability of evidence

On basis of the information provided in the selected papers and on knowledge of activities offered in the respective geographical areas, community-oriented activities were classified into three levels:

- Very extensive: participation of a broad spectrum of stakeholders, including the local municipality, primary care, civil society, media and local stores in collaboration.
- Extensive: active local municipality with established collaboration between stakeholders.
- Limited: ordinary public health work, including building of bicycle paths and key-hole marking of healthy food, which are standard in Swedish society.

If the community activities were classified as “very extensive”, one point was deducted due to possibly reduced transferability.

## Results

Although all the observational studies showed good design and control for main confounders, we downgraded all these studies one level because of risk of bias. The included studies showed high consistency for all outcomes. Two studies were downgraded one level because of lack of precision on some outcomes: physical activity, smoking and food intake [18] and systolic and diastolic blood pressure [12].

The included studies varied in follow-up time from 1 to 26 years (Table 1). They also varied in terms of the number of individual health dialogues offered (one to three) during the intervention period (Table 2). Also, the extent of the community activities in the intervention groups varied, ranging from very extensive activity in three studies [12, 13, 17] to limited activity in the RCT [18]. The community activities offered in the control groups were limited in all studies, except for Lingfors et al. 2009 [16]. A summary of the extent of interventions is listed in Table 2. The studies of Weinehall 1999 [12], Weinehall 2001 [13] and Lingfors 2019 [17] were downgraded one level because of risk of insufficient transferability/external validity when the community-oriented measures had been very extensive.

### Effects on outcome and level of evidence for the Swedish model of health dialogues

The results from the studies on each of the health outcomes are listed in Tables 3, 4, 5, 6, 7, 8, 9, 10, 11, 12, 13, 14, and 15.

**Table 3:** Outcome measure: all-cause mortality

Study	Results	Level of evidence	Downgrading according to GRADE*
Blomstedt et al. 2015 [14]	I (target group, ITT): SMR 90,6% (significant difference, 95% CI 88.2–93.0%) I (participants in health dialogue): SMR (%) 66.3% (significant difference, 95% CI 63.7–69.0%)	Moderate	Confounding −1
Lingfors et al. 2019 [17]	I (target group, ITT): Odds ratio 0.71 (significant difference, 95% CI 0.53–0.95) I (participants in health dialogue): Odds ratio 0.57 (significant difference, 95% CI 0.40–0.81)	Low	Confounding −1 Transferability −1

The study from Västerbotten region with moderate level of evidence was substantially larger than the study from Habo municipality

*I* = Intervention group, *ITT* Intention to treat, *C* Reference/control group, *CI* Confidence interval, *SMR* Standardized Mortality Ratio

^*^Reasons for downgrading: Confounding, Transferability and Precision (see methods)

**Table 4:** Outcome measure: cardiovascular mortality

Reference	Results	Level of evidence	Downgrading according to GRADE*
Blomstedt et al. 2015 [14]	I (target group, ITT): SMR 95.0% (significant difference, 95% CI 90.7–99.4%) I (participants in health dialogue): SMR (%) 68.9% (significant difference, 95% CI 64.2–73.9)	Moderate	Confounding −1

Only one study, but very large

*I* = Intervention group, *ITT* Intention to treat, *C* Reference/control group, *CI* Confidence interval, *SMR* Standardized Mortality Ratio

^*^Reasons for downgrading: Confounding, Transferability and Precision (see methods)

**Table 5:** Outcome measure: cholesterol

Reference	Results	Level of evidence	Downgrading according to GRADE*
Weinehall et al. 1999 [12]	Men: I: −0.82 mmol/L C: −0.07 mmol/l Women: I: −1.24 mmol/l C: −0,26 mmol/l (significant difference men and women merged, *p* < 0.001)	Low	Confounding −1 Transferability −1
Weinehall et al. 2001 [13]	Men: I: −0.83 mmol/l C: −0.26 mmol/l (significant difference, *p* < 0,001) Women: I: −0.73 mmol/l C: −0.9 mmol/l (significant difference, *p* < 0.001)	Low	Confounding −1 Transferability −1
Lingfors et al. 2009 [16]	Proportion with cholesterol > 5.0 mmol/l I: −2.5 percent units C: + 10,4 percent units (significant difference 99% CI)	Moderate	Confounding −1
Hellstrand et al. 2017 [18]	I: −0.2 mmol/L C: −0.1 mmol/L (no significant difference, *p* = 0.57)	High	
Eliasson et al. 2018 [15]	Decreased in both I and C (no significant difference, *p* = 0.9)	Moderate	Confounding −1

The assessment was based on five studies: One study with moderate level of evidence and two studies with low level of evidence showed effect, the latter large effect. Two studies show no effect, one of these recruited rather few participants and had limited community-oriented measures

*I* = Intervention group, *ITT* Intention to treat, *C* Reference/control group, *CI* Confidence interval, *SMR* Standardized Mortality Ratio

^*^Reasons for downgrading: Confounding, Transferability and Precision (see methods)

**Table 6:** Outcome measure: fasting P-Glucose

Reference	Results	Level of evidence	Downgrading according to GRADE*
Hellstrand et al. 2017 [18]	I: ± 0,0 mmol/L C: −0.1 mmol/L (no significant difference, *p* = 0.102)	High	
Eliasson et al. 2018 [15]	Decreased in I and increased in C (significant difference, *p* = 0.003)	Moderate	Confounding −1

One study with moderate level of evidence showed effect and one with relatively few participants and high level of evidence showed no difference

**Table 7:** Outcome measure: diabetes

Reference	Results	Level of evidence	Downgrading according to GRADE*
Eliasson et al. 2018 [15]	Prevalence: No significant difference in time trend (*p* = 0.5)	Moderate	Confounding −1

Only one study, moderately large

*I* = Intervention group, *ITT* Intention to treat, *C* Reference/control group, *CI* Confidence interval, *SMR* Standardized Mortality Ratio

^*^Reasons for downgrading: Confounding, Transferability and Precision (see methods)

**Table 8:** Outcome measure: Systolic blood pressure (SBP)

Reference	Results	Level of evidence	Downgrading according to GRADE*
Weinehall et al. 1999 [12]	Men: I: −8.5 mmHg (significant difference in time trend, *p* < 0,05) C: −0.2 mmHg (no significant difference) Women: I: −7.2 mmHg (significant difference in time trend, *p* < 0.001) C: −0.5 mmHg (no significant difference)	Very low	Confounding −1 Transferability −1 Precision −1 (no testing of difference between I and C)
Weinehall et al. 2001 [13]	Men: I: −6.7 mmHg C: −1.1 mmHg (significant difference, *p* < 0.01) Women: I: −7.3 mmHg C: −2.9 mmHg (significant difference, *p* < 0.05)	Low	Confounding −1 Transferability −1
Lingfors et al. 2009 [16]	Proportion with SBP ≥ 140 mmHg I: −3.7 percent units C: + 0.5 percent units (significant difference, 99% CI)	Moderate	Confounding −1
Hellstrand et al. 2017 [18]	I: −1.5 mmHg C: −1.0 mmHg (no significant difference, *p* = 0.74)	High	
Eliasson et al. 2018 [15]	Decreased more in I compared to C (significant difference, *p* = 0.04)	Moderate	Confounding −1

Four out of five studies showed effect, large effects in two studies. One study, with relatively few participants, showed no effect

*I* = Intervention group, *ITT* Intention to treat, *C* Reference/control group, *CI* Confidence interval, *SMR* Standardized Mortality Ratio

^*^Reasons for downgrading: Confounding, Transferability and Precision (see methods)

**Table 9:** Outcome measure: Diastolic blood pressure (DBP)

Reference	Results	Level of evidence	Downgrading according to GRADE*
Weinehall et al. 1999 [12]	Men: I: No significant difference in time trend C: No significant difference in time trend Women: I: No significant difference in time trend C: No significant difference in time trend	Very low	Confounding −1 Transferability −1 Precision −1 (no testing of difference between I and C)
Weinehall et al. 2001 [13]	Men: I: −3.1 mmHg C: + 0.4 mmHg (significant difference, *p* < 0,01) Women: I: −2.7 mmHg C: −2.2 mmHg (no significant difference, *p* > 0,05)	Low	Confounding −1 Transferability −1
Lingfors et al. 2009 [16]	Proportion with DBP ≥ 90 mmHg I: −7.7 percent units C: −4.4 percent units (significant difference, 99% KI)	Moderate	Confounding −1
Hellstrand et al. 2017 [18]	I: −0.3 mmHg C: ± 0.0 mmHg (no significant difference, *p* = 0.82)	High	
Eliasson et al. 2018 [15]	I: Decreased approximately 4 mmHg C: Increased approximately 2 mmHg (significant difference, *p* < 0,001)	Moderate	Confounding −1

Three out of five studies show effect (two with moderate and one with low level of evidence), one of these only for men. Large effect in one study. Two studies show no difference, one of those with high level of evidence and relatively few participants, and one with very low level of evidence

*I *Intervention group, *ITT* Intention to treat, *C* Reference/control group, *CI* Confidence interval, *SMR* Standardized Mortality Ratio

^*^Reasons for downgrading: Confounding, Transferability and Precision (see methods)

**Table 10:** Outcome measure: Body Mass Index (BMI)

Reference	Results	Level of evidence	Downgrading according to GRADE*
Weinehall et al. 1999 [12]	Men: I: significant difference in time trend (*p* < 0,05) C: No significant difference in time trend Women: I: No significant difference in time trend C: No significant difference in time trend	Very low	Confounding −1 Transferability −1 Precision −1 (no testing of difference between I and C)
Lingfors et al. 2009 [16]	Proportion with BMI ≥ 25 kg/m2 I: ± 0.0 percent units C: + 9.6 percent units (significant difference, 99% CI)	Moderate	Confounding −1
Hellstrand et al. 2017 [18]	I: −0.35 kg/m2 C: −0.05 kg/m2 (significant difference, *p* = 0.031)	High	
Eliasson et al. 2018 [15]	Increased in I and C (no significant difference, *p* = 0,8)	Moderate	Confounding −1

Two studies with high and moderate level of evidence, respectively, show positive effect, one study with moderate level of evidence shows no effect, and one study with very low level of evidence shows negative effect for men

*I *Intervention group, *ITT* Intention to treat, *C* Reference/control group, *CI* Confidence interval, *SMR* Standardized Mortality Ratio

^*^Reasons for downgrading: Confounding, Transferability and Precision (see methods)

**Table 11:** Outcome measure: waist circumference

Reference	Results	Level of evidence	Downgrading according to GRADE*
Lingfors et al. 2009 [16]	Proportion with increased waist circumference (men ≥ 94 cm, women ≥ 80 cm) I: + 2.1 and C: + 11.6 percent units (significant difference, 99% KI)	Moderate	Confounding −1
Hellstrand et al. 2017 [18]	I: −1.5 cm C: + 0.6 cm (significant difference, *p* ≤ 0.001)	High	
Eliasson et al. 2018 [15]	No significant difference, *p* = 0.2	Moderate	Confounding −1

Two studies with high and moderate level of evidence respectively, show positive effect, one study with moderate level of evidence shows no difference

*I *Intervention group, *ITT* Intention to treat, *C* Reference/control group, *CI* Confidence interval, *SMR* Standardized Mortality Ratio

^*^Reasons for downgrading: Confounding, Transferability and Precision (see methods)

**Table 12:** Outcome measure: physical activity

Reference	Results	Level of evidence	Downgrading according to GRADE*
Lingfors et al. 2009 [16]	Proportion with insufficient physical activity I: + 3.7 percent units C: + 0.5 percent units (no significant difference)	Moderate	Confounding −1
Hellstrand et al. 2017 [18]	Proportion with low physical activity I: −5 percent units (no significant difference, *p* = 0.052) C: −1,2 percent units (no significant difference, *p* = 0.855)	Moderate	Precision −1 (no testing of difference between I and C)
Eliasson et al. 2018 [15]	Physical inactivity decreased in I and C (no significant difference, *p* = 0.6)	Moderate	Confounding −1

No study shows a significant difference

*I *Intervention group, *ITT* Intention to treat, *C* Reference/control group, *CI* Confidence interval, *SMR* Standardized Mortality Ratio

^*^Reasons for downgrading: Confounding, Transferability and Precision (see methods)

**Table 13:** Outcome measure: smoking

Reference	Results	Level of evidence	Downgrading according to GRADE*
Weinehall et al. 1999 [12]	I: No significant difference in time trend C: No significant difference in time trend	Very low	Confounding −1 Transferability −1 Precision −1 (no testing of difference between I and C)
Weinehall et al. 2001 [13]	Men: I: No significant difference C: Significantly less smokers Women: I: No significant difference C: No significant difference	Very low	Confounding −1 Transferability −1 Precision −1 (no testing of difference between I and C)
Lingfors et al. 2009 [16]	I: −9.4 percent units C: −8.3 percent units (no significant difference)	Moderate	Confounding −1
Hellstrand et al. 2017 [18]	I: −0.6 percent units (no significant difference, p = 1,000) C: ± 0.0 percent units (no significant difference, *p* = 1,000)	Moderate	Precision −1 (no testing of difference between I and C)
Eliasson et al. 2018 [15]	Men: I: −13 percent units C: −8 percent units Women: I: −11 percent units C: −17 percent units (decreased significantly more in I, *p* = 0.01)	Moderate	Confounding −1

One study shows positive effect, three studies show no effect, and one study shows negative effect for men. Conflicting results, two of the studies have very low level of evidence

*I *Intervention group, *ITT* Intention to treat, *C* Reference/control group, *CI* Confidence interval, *SMR* Standardized Mortality Ratio

^*^Reasons for downgrading: Confounding, Transferability and Precision (see methods)

**Table 14:** Outcome measure: Dietary habits

Reference	Results	Level of evidence	Downgrading according to GRADE
Lingfors et al. 2009 [16]	Proportion with unhealthy eating habits I: −10.8 percent units C: −4.0 percent units (significant difference, 95% KI)	Moderate	Confounding −1
Hellstrand et al. 2017 [18]	Proportion with unhealthy eating habits I: −11.1 percent units (significant difference, *p* = 0.031) C: −.6 percent units (no significant difference, *p* = 0.860)	Moderate	Precision −1 (no testing of difference between I and C)

**Table 15:** Outcome measure: Alcohol consumption

Reference	Results	Level of evidence	Downgradingaccording to GRADE*
Hellstrand et al. 2017 [18]	Alcohol consumption (equivalent of hard liquor/week) I:—1,0 C:—4,0 (no significant difference, p = 0,167)	High	

One study with relatively few participants and high level of evidence has not shown effect

*I *Intervention group, *ITT* Intention to treat, *C* Reference/control group, *CI* Confidence interval, *SMR* Standardized Mortality Ratio

^*^Reasons for downgrading: Confounding, Transferability and Precision (see methods)

The effects on all-cause mortality were evaluated in two studies [14, 17], both with long follow-up times (10 and 26 years, respectively). One of these studies (from Västerbotten) [14] included 101 918 individuals (baseline average 48.9 years) and the second (from Habo) [17] included 757 individuals (baseline average 37.5 years). In the intention-to-treat analyses, both studies showed large and significant effects on risk for all-cause mortality (9% and 29% reduction, respectively) in relation to comparable populations or all of Sweden (Table 3). The level of evidence was found to be moderate (+ + +) (Table 16). For those who participated in the program, reduction of all-cause mortality was 34% and 43%, respectively. The participation in the health dialogues, was 59% [14] and 86% [17], respectively, in these two studies.

**Table 16:** Summary of results

Outcome measure	No of studies	Results summary: Health dialogues is associated with…	Level of evidence
Mortality			
Total mortality	2	Reduced risk of total mortality	+ + +
Cardiovascular mortality	1	Reduced risk of CVD-mortality	+ + +
Disease diagnosis			
Diabetes diagnosis	1	No effect on the prevalence of diabetes	+ +
Cholesterol	5	Reduced levels of p-cholesterol	+ +
Fasting P-Glucose	2	Reduced levels of fP-Glucose	+ +
Systolic blood pressure (SBP)	5	Reduced SBP	+ +
Diastolic blood pressure (DBP)	5	Reduced DBP	+ +
Body mass index (BMI)	4	Reduced BMI	+ +
Waist circumference (WC)	3	Reduced WC	+ + +
Lifestyle behaviours			
Physical activity (PA)-level	3	No effect on PA-level	+ + +
Smoking	5	No effect on smoking	+ +
Dietary habits	2	Improved dietary habits	+ + +
Alcohol consumption	1	No effect on alcohol consumption	+ + +

All other abbreviations explained in the table

*CVD* Cardiovascular, *fP-Glucose* fasting plasma-Glucose

Risk of CVD mortality, which was only examined in one study [14], was reduced by 5% in the intention-to-treat analysis and 31% in the per protocol analysis (Table 3)*.*

The Swedish model was also associated with reduced levels of CVD risk factors, with varying level of size of effect and evidence, as shown in Tables 5, 6, 7, 8, 9, 10, 11, 12, 13, 14, and 15 and summarized in Table 16. The following significant effects (levels of evidence) were reported: reduced levels of serum cholesterol (+ +), fasting blood glucose (+ +) and systolic (+ +) and diastolic blood pressure (+ +); large effects were observed for both measures of blood pressure. In addition, lower BMI (+ +), reduced waist circumference (+ + +) and improved dietary habits (+ + +) were reported. No effects were found on the prevalence of diabetes, physical activity level, alcohol consumption or smoking (Tables 7, 12, 14, and 15).

## Discussion

This paper presents the first systematic review of the Swedish model of health dialogues, a combined individual- and community-based primary preventive program of CVD. Seven studies fulfilled the inclusion criteria, six of these were observational studies and one RCT. Study results showed that the program was associated with large reductions of total and CVD mortality (moderate level of evidence) and significant reductions of systolic and diastolic blood pressure, and serum cholesterol, fasting blood glucose, BMI and waist circumference (moderate to low level of evidence). Regarding health behaviors, only dietary habits showed significant positive effects.

All but one of the included studies were observational, i.e. NRCTs. Although RCT is the gold standard in clinical settings, this design is not feasible for population-based public health interventions, especially where community intervention is a central part. According to the World Health Organization, an RCT could in this case even give misleading results [20]. All six observational studies presented data that controlled for effects of possible confounders. However, as residual confounding could not be ruled out (e.g. for psychosocial factors), these studies were all downgraded one level because of risk of bias. Therefore, these studies could not be upgraded in level of evidence despite large treatment effects for all-cause mortality [14, 17], blood pressure [12, 13, 15] and cholesterol [12, 13, 16]; thus, the grading should be regarded as conservative. The included studies showed high consistency of results.

External validity and transferability of results are important, especially when evaluating population-based studies. A key question for transferability is the extent of the intervention, especially regarding community activities and the local context for these interventions. The extent of community activity varied between included studies. Two studies [12, 17], evaluated interventions performed in rather small local communities (Norsjö and Habo) in the early phase of the development of the Swedish model of health dialogues**.** Both describe extensive community interventions. These included new models for support of physical activity and healthy diet, as exemplified by “shop for life”, where grocery stores were inspired to adjust prices according to health effects (e.g. lower cost for vegetables and higher for snacks), public involvement in study groups, mass media campaigns, and the development of “Keyhole” markings of healthy food. This Keyhole symbol was developed in Norsjö by combining an old symbol for the community (a heart) with the food pyramid; thus forming a keyhole. The Keyhole symbol was registered by The Swedish Food Agency as a trademark and the labelling system enforced through a regulation. The symbol aims to help consumers identify healthier options when buying food, and also aims to stimulate food producers to develop healthier products, for 32 food groups.

This level of community support and intervention was not described in the later studies. However, interventions to promote healthy behaviors have over time been developed all over the country, and include smoking bans in restaurants, building of bicycle paths and outdoor gyms. The Keyhole marking of food is today routine in Sweden. These societal changes would potentially aid future implementation of the Swedish model.

Another factor influencing transferability is the composition of the intervention group. It is well known that groups with lower SES have higher premature mortality and show more unhealthy behaviors [21]. Therefore, a key question is if interventions reach all SES groups, not only those with higher SES, which could further increase inequalities in health. In their study, Blomstedt et al. 2015 [14] showed that those with shorter education participated to a greater extent in the health dialogues and more life years were saved in the middle and low SES groups than in the high SES group. Lingfors et al. 2019 [17] showed that the intervention population had lower SES, but better results in terms of survival, and Weinehall [13] showed that cholesterol levels after intervention were better among people with shorter education. This suggests the studied intervention also reached those with lower SES, thereby possibly reducing inequities in health.

No publication bias could be identified. Some additional studies on the Swedish model [22–24], supporting the findings of the present review, did not fulfil the inclusion criteria and was thus not included in our data.

The combined individual- and community-based primary preventive program of CVD reviewed here is created and applied in the Swedish context and there are no identical international studies for comparison. It is also important to note that general health checks, which are well known not to have any effect on all-cause or CVD mortality [7], differ in key areas from the Swedish model. They focus on screening for early detection of risk factors and latent disease, with few cases encompassing interventions of behavioral change, are seldom integrated in primary care, and none includes community interventions.

Some similarities do exist between the studies in our review and studies included in the Cochrane report from 2011 [8]; these studies focused on advice and education on CVD risk factors among individuals with diabetes and hypertension and showed effects on risk factors and subsequent CVD morbidity and all-cause mortality. However, both reviews focus on individuals at risk, i.e. high-risk interventions, whereas the Swedish model were population based and focused on all participants within a given geographical area and this intervention was done in combination with community activities. Positive findings regarding CVD events were found in a systematic review from the US Preventive Services Task Force [25], showing that counselling on behavioural change regarding physical activity and diet may have a short-term (6–12 months) effect, also in those without CVD, but long-term effects are insufficiently studied. Another systematic review from US [26], analyzed 36 community multi-component intervention programs performed 1970–2008. These included mass media campaigns, screening combined with interventions for behavioral change and community activities. Authors conclude that the studies showed promising results on CVD mortality and risk factors, but more studies are needed to confirm their findings.

The results from the Swedish model showed the strongest effect and evidence for all-cause and CVD mortality and for effect on biological risk factors. However, except for effects on eating habits, they did not show evidence for effect on the individuals’ health behaviors, which is a vital part of this preventive program. This may seem like a paradox, given the wealth of evidence on the association of poor health behaviors with CVD risk factors, adverse events and mortality. This may imply that part of the effect on mortality is conveyed via other CVD risk factors e.g. psychosocial factors. However, another explanation for these findings could be a result of methodological limitations and differences, such as less precise measures of health behaviors (self-report) compared to blood tests and hard endpoints such as mortality. A notable exception is for effects seen on food intake. While examined in only two studies, the proportion of individuals with unhealthy eating habits decreased in a similar way in both studies [16, 18] and in the larger study [16] the reduction was 10.8% in the intervention area compared to 4.0% in the reference area. One possible reason for these differences in effects on health behaviors is that food intake was a central part in these health dialogues.

The Swedish model reviewed here is a multi-factorial intervention. Few studies have been designed to distinguish which component of their total intervention has the greatest effect. One study by Weinehall [12] tried to delineate the community part from the health dialogue. This showed that community intervention alone was associated with a reduction in cholesterol. When including the health dialogue, the effect occurred faster but was not greater. One of the excluded studies (no control group), was dominated by community intervention, which was associated with reduced CVD mortality [22]. Finally, in two studies by Lingfors [16] and Hellstrand [18], the intervention and control groups received the same level of community intervention, and the individual health dialogue (alone) was associated with effect on CVD risk factors. Therefore, while it is not clear how large the community intervention needs to be, or which parts of the health dialogue are necessary for effect, these results indicate that both these major components need to be included in future population-based primary prevention interventions.

The clinical implications of the present review of a combination of individual health dialogues and community intervention are large. The main findings, showing that the program has substantial effects on both CVD and all-cause mortality, indicate that effective primary preventive interventions should include both individual and community-based components. In 2025 this concept is offered in 13 out of 21 regions in Sweden. Although, the validity of these results in different health settings, outside the Swedish context is difficult to ascertain, the general principles should be applicable. Indeed, the main difference from previous reviews and reports on general health checks (which showed no effect or unclear results) is that the Swedish model is a multifactorial intervention where both the health dialogue and community components are included. While the integration of the Swedish model in primary care may offer a systematic structure for primary prevention planning and intervention in similar health settings to the Swedish system, also other settings may benefit from the results, highlighting the need for a combination of health dialogues and community-based interventions.

## Conclusion

The results of this first systematic review of the Swedish model of health dialogues show that a combined individual- and community-based primary preventive program of CVD is associated with reduced CVD and all-cause mortality (moderate level of evidence). The model was also associated with reduction of biological CVD risk factors and improvement of diet (moderate level of evidence). The Swedish model is a multifactorial intervention that includes both individual and community/societal intervention. While the respective contributions of both parts of the intervention cannot be delineated, the results of this study indicate that both individual and communal intervention must be included.

## Data Availability

No datasets were generated or analysed during the current study.
