# Correction: The Swedish model of health dialogues, a combined individual- and community-based primary preventive program for cardiovascular disease, is associated with reduced mortality: a systematic review

**DOI:** 10.1186/s12889-026-26306-7

**Published:** 2026-01-28

**Authors:** Mats Borjesson, Margareta Kristenson, Lars Jerdén, Yvonne Forsell

**Affiliations:** 1 Institution of Medicine, Department of Molecular and Clinical Medicine, Sahlgrenska Academy, University of Gothenburg, Gothenburg, Sweden; 2 Department of Medicine, Geriatrics and Emergency Care, Center for Lifestyle Intervention, Sahlgrenska University Hospital, Gothenburg, Sweden; 3 Department of Health, Medicine and Caring Sciences, Linköping University, Linköping, Sweden; 4 Center for Clinical Research Dalarna-Uppsala University, Uppsala, Sweden; 5 Department of Global Public Health, Karolinska Institute, Stockholm, Sweden

**Correction: BMC Public Health 25, 3288 (2025)**

**https://doi.org/10.1186/s12889-025-24353-0**

Following publication of the original article [1], the authors identified an error regarding the presentation of all author names. Specifically, the given names and family names were transposed.

Incorrect: Borjesson Mats, Kristenson Margareta, Jerdén Lars and Forsell Yvonne.

Correct: Mats Borjesson, Margareta Kristenson, Lars Jerdén and Yvonne Forsell.

The author group has been updated above and the original article [1] has been corrected.
